# The moderating effect of perceived organizational support on presenteeism related to the inclusive leadership

**DOI:** 10.1186/s12912-024-01816-0

**Published:** 2024-02-24

**Authors:** Ting Wang, Hui Qin, Ziqi Zhang, Yonghao Qin

**Affiliations:** 1 Nursing Department, The First Affiliated Hospital of Soochow University, Suzhou, Jiangsu Province, China; 2 Jingchu University of Technology, Hubei Province, Jingmen, China; 3 School of Nursing, Soochow University, Suzhou, Jiangsu Province, China

**Keywords:** Presenteeism, Perceived organizational support, Inclusive leadership, Clinical nurse

## Abstract

**Aim:**

This study aimed to assess inclusive leadership and presenteeism among clinical nurses and to examine the moderating effect of perceived organizational support on presenteeism related to the inclusive leadership among nurses.

**Background:**

Nurses’ presenteeism has become common. In hospitals, inclusive leadership is an acknowledged leadership style that has a positive influence on nurses. However, little emphasis has been paid to research on their relationships and moderating effect.

**Methods:**

A cross-sectional study was undertaken to assess 2222 nurses using a general information questionnaire, Stanford Presenteeism Scale (SPS-6), Perceived Organisational Support Scale, and Inclusive Leadership Scale. Study variables were analyzed using descriptive statistics, correlation, and structural equation modelling (SEM).

**Results:**

Presenteeism was relatively severe among clinical nurses. There were correlations between inclusive leadership, perceived organizational support and presenteeism. Perceived organizational support moderated the relationship between inclusive leadership and presenteeism.

**Discussion and conclusion:**

Nursing managers should actively adopt an inclusive leadership style and improve nurses' sense of perceived organizational support to improve clinical nurses' presenteeism behaviors.

**Implications for nursing policy and practice:**

Healthcare organizations and nursing managers should pay attention to the psychological needs of their nurses, provide complete understanding and support, encourage staff to actively participate in their work and contribute new ideas and opinions, reduce the incidence of presenteeism, and improve nurses' sense of well-being at work.

**Supplementary Information:**

The online version contains supplementary material available at10.1186/s12912-024-01816-0.

## Introduction

Clinical nurses have huge responsibilities with complex working environments and objects; their physical and mental health conditions are often affected to varying degrees, resulting in a rise in presenteeism [1, 2]. Presenteeism refers to the phenomenon of people, despite poor health conditions that should prompt rest and absence from work, still turning up at their jobs, resulting in a decrease in concentration and inability to devote themselves to their work, thus causing a decrease in work efficiency and inability to perform duties [3]. Moreover, it affects the health and safety outcomes of patients, the financial profitability of the organization, and the quality of nursing services [4]. Enhancing the presenteeism behavior of clinical nurses has become an urgent issue for nursing managers.

Robert Eisenberger, an American social psychologist, found that employees are more inspired and motivated to perform well when they perceive perceived organizational support (POS), which pertains to the employees' perception of the organization's regard for their well-being and contribution [5]. When employees perceive such support, they are more likely to feel valued and supported by the organization. The POS has a direct impact on the presenteeism behaviors of nurses, according to additional findings. The more job support nurses receive, the lower the frequency of presenteeism and the lower the resulting loss of production [6, 7].

In leadership studies, inclusive leadership belongs to the leadership style based on respect, recognition, responsiveness, and responsibility between leaders and employees, manifesting an interactive atmosphere of openness, effectiveness, and accessibility in daily interactions with employees. Inclusive leadership is a new type of willingness to adopt new ideas and concepts from employees during the work process and encourages active participation in work management and decision-making [8]. Inclusive leadership styles have the potential to effectively prevent or mitigate the incidence of presenteeism, thereby improving the overall productivity of clinical nurses.

According to the principles of social exchange theory, inclusive leaders, through their supportive and caring leadership style, can foster a positive psychological state and organizational commitment among nurses. Meanwhile, a significant positive correlation was found between inclusive leadership and perceived organizational support [9]. Consequently, we hypothesize that inclusive leadership’s initial impact will be perceived organizational support for nurses. This, in turn, is expected to may reduce a range of negative work behaviors such as presenteeism. Thus, this study aims to investigate the moderating role of perceived organizational support between inclusive leadership and presenteeism by investigating the current situation of clinical nurses' presenteeism in four tertiary hospitals in Suzhou and serve as a reference for better management and intervention of clinical nurses' presenteeism.

## Methods

### Design and participants

In four tertiary institutions in Suzhou, China, 2253 registered nurses were selected for the study via convenience sampling. Inclusion criteria: (a) possessing a nurse practice certificate and being on duty; (b) having more than one year of clinical nursing experience; (c) providing informed consent to participate in this study. Nurses who were on vacation and came to the hospital temporarily to study and train were excluded. A total of 2253 questionnaires were distributed, 2253 questionnaires were returned, 31 with logical errors were eliminated, and 2222 questionnaires were effectively returned, with a valid return rate of 98.81%.

### Ethical considerations

The study was approved by the Ethics Committee of the First Affiliated Hospital of Soochow University. The consent of nurses who completed the electronic survey was deemed to be informed. Participants completed the electronic questionnaire anonymously, and their personal information was kept confidential.

## Measures

### Demographic information

Demographic information includes gender, age, highest education, marital status, children's status, appointment mode, title, position, years of service, department, night shift status, after-hours physical exercise, history of underlying diseases, health status, health status, and religious beliefs.

### The Stanford Presenteeism Scale (SPS-6)

The SPS-6 was developed by Koopman [10] and revised by Chinese scholar Zhao Fang [11]. The scale consists of 6 entries and is scored using a Likert 5-point scale, where entries 1 to 4 are scored positively, and entries 5 and 6 are scored negatively. The total scale score ranges from 6 (lower presenteeism) to 30 (higher presenteeism). Cronbach's α for the scale ranged from 0.76–0.90, indicating its good reliability and validity, and nurses have extensively used it. In this study, Cronbach's α for the scale was 0.884.

### Inclusive Leadership Scale

Carmeli et al. [12] developed the Inclusive Leadership Scale and translated and modified by Peng Wei and others [13]. The interactive process of the scale reflects three dimensions of openness, availability, and accessibility. The Likert 5 scale assigns scores from 1 (strongly disagree) to 5 (strongly agree). Higher scores indicate that the nurse perceives a more prominent inclusive leadership style. Cronbach's α for the scale was 0.90, indicating high reliability. In this sample, Cronbach's α for the scale was 0.856, which had a high internal consistency.

### Sense of perceived organizational support scale

This scale, developed by Hongmei Zuo [14], is based on the perceived organizational support scale developed by Chen Zhixia [15] and incorporates characteristics of the nursing profession, extensive consultation with experts, and appropriate modifications to certain items. The scale measured nurses’ perceived organizational support based on “emotional support” and “instrumental support”. The 5-point Likert scale of instrumental support was used to assign scores from 1 (strongly disagree) to 5 (strongly agree), and the higher the score, the stronger the sense of perceived organizational support. Cronbach's α for the scale was 0.90, widely used in the nursing community. In this study, Cronbach's α for the scale was 0.897.

### Data collection

Data was collected via survey questionnaires for one month, from June to July 2022. The survey followed the principles of anonymity and voluntariness. Participants were informed that they had the right to withdraw at any time. This study created an anonymous internet questionnaire using the Questionnaire Star platform. Before the official survey, the relevant heads of the nursing departments of the four hospitals received a thorough explanation of the study's objectives to secure their participation and permission. The researcher obtained the contact information of the head nurse of each hospital department through the relevant head of the nursing department and pinned the QR code or link of the questionnaire to the heads of each department. The head nurse then forwarded it to the Ding Talk of her department to inform the clinical nurses of her department to complete the questionnaire. To ensure the completeness and validity of the questionnaire, we used unified guidelines, set all questions as mandatory questions, each IP address could only be filled in once, and set the cumulative answer time to not less than 200s.

### Data analysis

The SPSS 27.0 software package was used for the statistical description of the data, and the mean ± standard deviation of the measured data was expressed as (x±s). The Pearson correlation coefficient was used to measure the correlation between clinical nurses' perceived organizational support, inclusive leadership, and presenteeism. The AMOS 24.0 software tool was used to construct structural equation models (SEM) , the χ^2^/df value must remain below 3; both the CFI and TLI had to attain a minimum threshold of 0.90 or higher. Furthermore, a favorable model fit was indicated by an RMSEA value below 0.08 [16].

## Results

### Demographic characteristics

Supporting information Table 1 (the end of the document text file) presents the demographic characteristics of all study variables. Of the 2222 clinical nurses, 94 were male, and 2128 were female. Among them, the presenteeism scores for the presence of children, years of employment, age, marital status, title, position, highest education, mode of appointment, night shift status, and department were statistically significant (*p* <0.05). In contrast, differences in gender, after-hours physical activity, history of underlying disease, and religious affiliation were not found to be statistically significant (*p* >0.05).

**Table 1:** Scores of clinically presenteeism behavior by demographic characteristics(*n*=2222)

**Projects**	**n**	**Presenteeism**	**Statistical quantities**	**P**
**Sex**			t=1.536	0.125
Man	94	17.46±2.50		
Woman	2182	16.93±3.30		
**Age**			F=15.462	0.000
20-30	957	17.78±2.37		
31-40	863	17.55±3.09		
41-50	304	14.41±3.62		
51-60	95	11.46±1.87		
>61	3	11.00±1.00		
**Academic qualifications**			F=15.624	0.000
Technical secondary school	21	13.09±3.37		
Universities and colleges	352	17.61±2.83		
Undergraduate course	1807	16.88±3.31		
Master degree or above	42	16.29±3.36		
**Marriage**			F=25.437	0.000
Unmarried	502	17.85±2.64		
Married	1631	16.71±3.36		
Divorced or widowed	89	16.33±4.03		
**Children**			t=8.827	0.000
With	1628	16.61±3.44		
Without	594	17.88±2.57		
Appointment Method				
Formal establishment	517	14.94±4.11	F=169.07	0.000
Contract system	1025	17.15±2.83		
Personnel Agency	680	18.17±2.34		
**Title**			F=309.33	0.000
Nurse	218	17.71±2.53		
Primary nurse	1200	17.58±2.81		
Nurse-in-charge	598	17.33±3.04		
Deputy director, nurse and above	206	11.39±1.35		
**Position**			F=49.73	0.000
Nurse	2103	17.14±3.17		
Head nurse	97	13.24±3.30		
Head of the nursing department	7	13.43±3.95		
Part-time college nursing teacher	15	16.47±2.75		
**Working years**			F=169.13	0.000
< 10 years	1336	17.72±2.75		
10~20	563	17.02±2.83		
21~30	184	15.35±4.23		
31~40	135	11.41±1.59		
>40	4	10.75±0.96		
**Night shift situation**			F=214.10	0.000
No night shift	384	13.66±2.57		
1~5 night shifts per month	1177	17.35±2.92		
6~10 night shifts per month	608	18.06±2.36		
> 10 night shifts per month	53	19.19±1.97		
**Administrative or technical offices**			F=3.64	0.000
Hematology department	290	17.29±3.19		
Operating room	139	16.19±3.23		
Emergency department	119	17.57±3.07		
Orthopaedics	118	16.92±2.98		
Outpatient internal medicine	90	14.67±3.76		
Respiratory medicine	86	17.53±3.09		
Department of critical care medicine	85	17.56±2.74		
General surgery	84	17.19±3.25		
Cardiovascular internal medicine	83	17.17±3.12		
Maternity department	80	16.64±3.14		
Oncology department	72	16.94±3.06		
Urology	69	17.07±3.15		
Brain surgery	69	17.46±2.85		
Cardiovascular surgery	58	16.72±2.87		
Outpatient surgery	55	15.00±3.76		
Department of gynecology	52	16.87±3.06		
Neurosurgery	50	17.30±2.88		
Other	623	16.83±3.56		
**Physical exercise after work (sweating or > 30 minutes)**			F=1.852	0.136
No	336	17.21±3.26		
1~2 times a month	1316	16.97±3.27		
Once or twice a week	350	16.63±3.22		
> 2 times a week	220	16.96±3.39		
**History of basic diseases**	1983	16.92±3.28	F=0.448	0.872
Without	15	17.07±2.87		
Heart disease	67	17.16±3.26		
Hypertension	16	16.75±3.07		
Diabetes	15	17.73±3.20		
Bronchial diseases	90	17.14±3.64		
Chronic gastritis	33	17.55±2.63		
Other musculoskeletal diseases	3	18.00±3.00		
History of trauma	1983	16.92±3.28		
**Physical condition**			t=0.681	0.496
Better	1859	16.97±3.30		
Discrepancy	363	16.84±3.14		
**Religious belief**			t=0.964	0.335
Without	2194	16.96±3.28		
With	28	16.36±2.89		

### Inclusive leadership, perceived organizational support, and level of presenteeism among clinical nurses

The results of the inclusive leadership behaviours, perceived organizational support, and nurses’presenteeism are as follows: (16.95±3.27) for presenteeism. (50.17±7.64) for total perceived organizational support; (38.69±6.14) for emotional support and (11.48±2.14) for instrumental support. (36.14±5.02) for total inclusive leadership; (12.23±2.01) for openness, (15.59±2.59) for availability and (8.32±5.02) for accessibility.

### Descriptive and correlational statistics

Additional file 1 (An additional movie file shows this in more detail) depicts the descriptive statistics and correlation matrix for all study variables. The result of the Pearson correlation analysis is consistent with our expectations. Specifically, negative correlations were noted not only between presenteeism and overall inclusive leadership (*R*-value = -0.333 and p-value <0.01), but also with all subscales of inclusive leadership, including openness (*R*-value = -0.280 and p-value <0.01), availability (*R*-value = -0.291 and p-value <0.01), accessibility (*R*-value = -0.315 and p-value <0.01). Furthermore, a positive correlation was found between overall inclusive leadership and overall perceived organizational support (*R*-value =0.600 and p-value <0.01). A negative correlation was also found not only between presenteeism and overall perceived organizational support (*R*-value = -0.310 and p-value <0.01) but also with all subscales of perceived organizational support which are emotional support (*R*-value = -0.275 and *p*-value <0.01)、instrumental support (*R*-value = -0.316 and *p*-value <0.01).

### Common bias method

Single Harman factor analysis was used to examine the bias of common methods in SPSS [17]. A total of six common factors with eigenvalues >1 were extracted, and the variance explained by the first common factor was 32.52%, which was less than 40% of the critical value. Thus, no significant deviation from the common method bias was found in this study.

### Moderation analyses

**Fig. 1:**
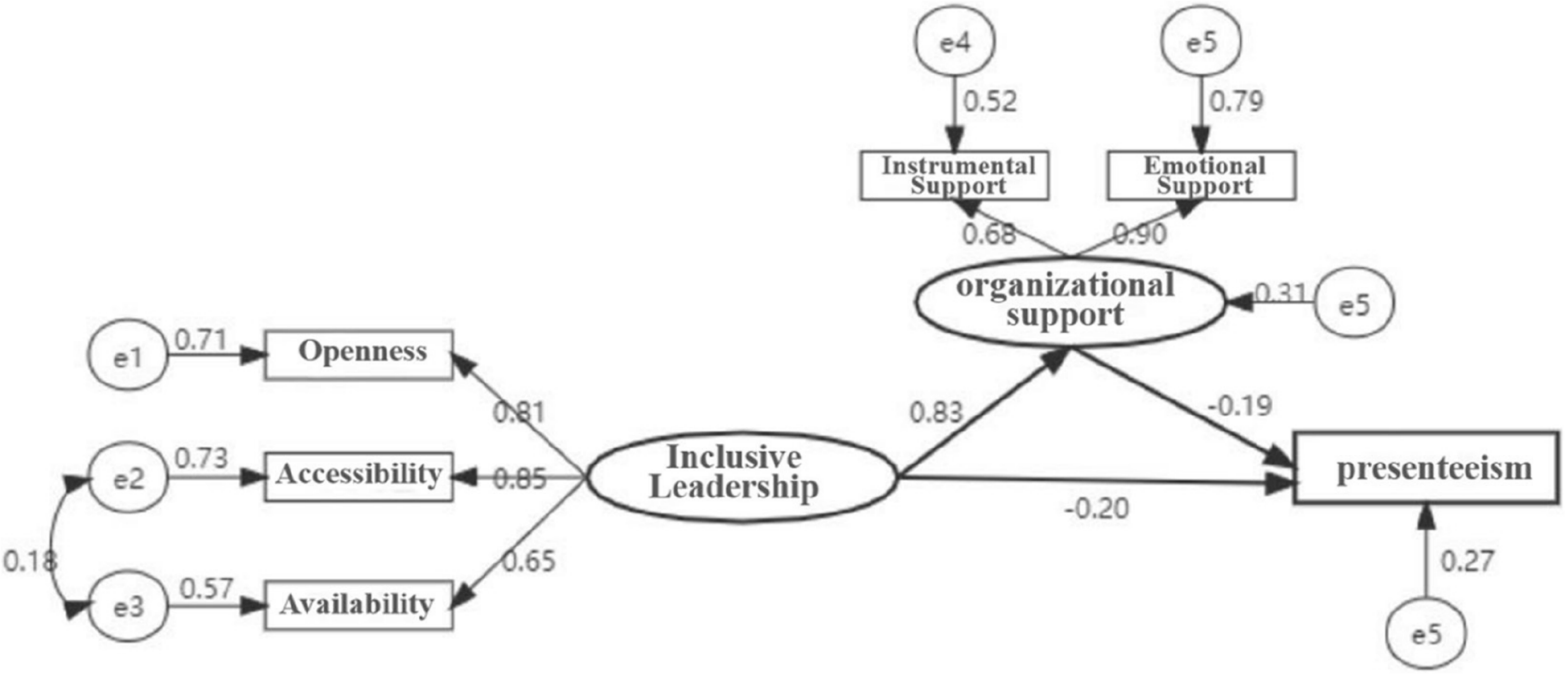
Impact of inclusive leadership behaviour on presenteeism: the moderating role of perceived organizational support

Structural equation modeling (SEM) showed that perceived organizational support have a moderating effect on the relationship between presenteeism and inclusive leadership. The model is presented in Fig. 1, which had very superior numbers of each fitted index: X^2^ /df was 1.410, <3.00; GFI= 0.989, CFI=0.988, AGFI=0.968, TLI=0.974 (all >0.9), and RMSEA=0.065 (<0.08), indicating that the structural equations fit well and the model is more reasonable.

## Discussion

The present study investigated the relationship between inclusive leadership and presenteeism, as well as the function of perceived organizational support as a moderator. First, the survey results in tertiary hospitals showed the presenteeism of nurses was relatively serious, consistent with Shdaifat’s research [18]. This analysis may be related to the high demand for medical care and the shortage of nursing human resources in the city. Due to the high workload, patients in both inpatient and outpatient departments of tertiary general hospitals are mobile, and the need for immediate emergency treatment and surgical resuscitation is very common. Besides, nursing is a profession where high pressure is associated. In environments that are rapidly changing and demanding, nurses must deliver high-quality care for patients. Therefore, nurses are susceptible to elevated levels of psychological stress and are consequently regarded as working in a high-risk, high-pressure environment [19]. Under continuous exposure to medical disputes and medical injuries, nurses' sense of occupational safety has declined. Therefore, hospital managers should pay proper to the physical and mental health of clinical nurses by implementing humanistic care, increasing the investment in the nursing profession, and providing targeted resources for violence response. These will improve nurses' motivation, inventiveness, and work efficiency, and reduce productivity loss caused by presenteeism behaviors.

Second, inclusive leadership, perceived organizational support, and presenteeism were all negatively correlated, i.e., the higher the nurses' perceived level of inclusive leadership, the lower their presenteeism, consistent with the research by Jin Y et al. [20]. Leadership styles impact clinical nurses, patient experience, work quality, and work environment [21]. According to Carmeli et al. [8], inclusive managers are more open to hearing employees' innermost thoughts from various points of view. They also encourage employees to speak up, advance their knowledge, and strengthen their sense of belonging at work. Finally, they can provide timely and efficient assistance to their staff. Under inclusive leadership, nurses will feel cared for and respected. With full support and understanding, they will be more inclined to produce better work performance and more conducive to the diversity of the work team [22]. Furthermore, it was found that nurses' perceived organizational support was negatively correlated to presenteeism, i.e., the higher the nurses' perceived organizational support, the lower their presenteeism behavior. According to social exchange theory [23], nurses who receive perceived organizational support can generate a positive psychological state and reduce negative emotions induced by work stress, thus reducing presenteeism behavior. Moreover, nurses who perceive a high level of perceived organizational support are likelier to demonstrate extra-role behaviour and job satisfaction, leading to increased productivity [24, 25]. Managers can improve nurses' presenteeism while strengthening the management of nurses by adopting an open and approachable leadership style, voluntarily providing them with a boost based on the basic principle of equality and reciprocity, giving them emotional and instrumental support, and accepting their shortcomings and suggestions.

Third, the relationship between inclusive leadership and presenteeism is mediated by perceived organizational support. Inclusive leaders can directly predict the presenteeism behaviors of nurses, and these behaviors can indirectly influence nurses through the organizational sense of support. The inclusive leadership style of nursing managers can shape the individuals perception (i.e., psychological safety and psychological engagement) about the organization context in a way that encourages helpfulness [26]. Conversely, under an inclusive leadership style, nurses can feel the support and help from their managers, which helps them to talk about their problems and reduces their emotional burnout, allowing them to be more actively involved in nursing work and improving the quality of their work [27]. Furthermore, nursing managers with an inclusive leadership style will take the initiative to empower nurses and actively adopt their innovative work suggestions to give full play to their initiative, making them feel respected and cared for, thereby improving the rigid work schedule and environment [28]. Clinical nurses work in a special environment with complex service targets and high work intensity. From the perspective of perceived organizational support, work and personal resources will inevitably be depleted, and perceived organizational support can be used as a protective factor to provide emotional and instrumental support for nurses and safeguard their physical and mental health. It has been found that when hospital organizations provide clinical nurses with obvious support and assistance, it stimulates good working conditions and the spirit of nurses, reducing job burnout [29]. Furthermore, perceived organizational support enhances nurses' sense of professional identity and professional value and improves job satisfaction [30]. Additionally, it promotes nurses' engagement in their work and reduces the occurrence of presenteeism behaviors. Therefore, it is necessary to pay close attention to the moderating variable of the sense of perceived organizational support to maximize the negative effect of inclusive leadership on presenteeism in nurses.

### Limitations

Inclusive leadership acknowledges and values the inherent worth of nurses. It involves working with others and fostering shared responsibility [31]. Therefore, the current study explored only one inclusive leadership, and Future research could examine other leadership styles.

This study had criteria for selecting sample measurements and study materials to some extent. The sample was selected from the nurses of only four tertiary hospitals in Suzhou, and its representativeness was somewhat constrained. Future research studies can choose to administer measurements in different areas, levels, and types of hospitals to verify the reliability of the study results. Furthermore, this study is a cross-sectional survey, arguing the causes of causality are insufficient. Thus, it is recommended that a follow-up longitudinal survey be conducted to demonstrate further the scientificity and validity of the study in terms of key variable characteristics.

### Implications for nursing policy and practice

The findings of this study demonstrate inclusive leadership can reduce presenteeism. Therefore, It is suggested that nurse managers give nurses full understanding and respect, conduct objective, thorough, and fair evaluations of the overall performance of clinical nurses in hospitals, and practice more praise and less punishment to alleviate negative work emotions by focusing on the lives of nurses and attach importance to their professional development. Establish a harmonic and cordial relationship with them by giving fair advancement possibilities, carrying out career development training, encouraging participation in continuing education programs, etc.

Moreover, the finding that perceived organizational support moderated the relationship between inclusive leadership and nurses’ presenteeism behavior suggested paying attention to the internal mechanism by which inclusive leadership influences presenteeism behavior. Nurse managers should encourage nurses to express their thoughts and suggestions and provide timely perceived organizational support, such as opportunities and resources to reduce presenteeism.

## Conclusions

This study found that the present situation of presenteeism in clinical nurses in Suzhou is serious, both inclusive leadership and perceived organizational support have a negative predictive effect on presenteeism behavior. Thus, to reduce nurses' presenteeism behavior and improve the overall work quality, healthcare institutions must cultivate a positive and supportive leadership culture, inclusive leadership is a great choice. Besides, the perceived organizational support partially mediates the relationship between inclusive leadership and presenteeism to some extent. That is, when nurses perceived that leaders showed more inclusiveness in their performances, thoughts and suggestions, they perceived being more valued and supported by the organization, reducing their presenteeism behavior. Thus, nursing managers should offer more emotional and practical support to nurses to reduce presenteeism.

### Supplementary Information

**Supplementary Material 1.**

## Data Availability

The datasets used and analyzed during the current study are available from the corresponding author upon reasonable request.
